# Metabolomics reveals the response of hydroprimed maize to mitigate the impact of soil salinization

**DOI:** 10.3389/fpls.2023.1109460

**Published:** 2023-06-07

**Authors:** Enying Zhang, Xingjian Zhu, Wenli Wang, Yue Sun, Xiaomin Tian, Ziyi Chen, Xinshang Mou, Yanli Zhang, Yueheng Wei, Zhixuan Fang, Neil Ravenscroft, David O’Connor, Xianmin Chang, Min Yan

**Affiliations:** ^1^ College of Agronomy, Qingdao Agricultural University, Qingdao, China; ^2^ School of Agriculture, Food and Environment, Royal Agricultural University, Cirencester, United Kingdom; ^3^ International Agriculture University, Tashkent, Uzbekistan

**Keywords:** land degradation, soil salinization, hydropriming, progesterone, metabolomics analysis

## Abstract

Soil salinization is a major environmental stressor hindering global crop production. Hydropriming has emerged as a promising approach to reduce salt stress and enhance crop yields on salinized land. However, a better mechanisitic understanding is required to improve salt stress tolerance. We used a biochemical and metabolomics approach to study the effect of salt stress of hydroprimed maize to identify the types and variation of differentially accumulated metabolites. Here we show that hydropriming significantly increased catalase (CAT) activity, soluble sugar and proline content, decreased superoxide dismutase (SOD) activity and peroxide (H_2_O_2_) content. Conversely, hydropriming had no significant effect on POD activity, soluble protein and MDA content under salt stress. The Metabolite analysis indicated that salt stress significantly increased the content of 1278 metabolites and decreased the content of 1044 metabolites. Ethisterone (progesterone) was the most important metabolite produced in the roots of unprimed samples in response to salt s tress. Pathway enrichment analysis indicated that flavone and flavonol biosynthesis, which relate to scavenging reactive oxygen species (ROS), was the most significant metabolic pathway related to salt stress. Hydropriming significantly increased the content of 873 metabolites and significantly decreased the content of 1313 metabolites. 5-Methyltetrahydrofolate, a methyl donor for methionine, was the most important metabolite produced in the roots of hydroprimed samples in response to salt stress. Plant growth regulator, such as melatonin, gibberellin A8, estrone, abscisic acid and brassinolide involved in both treatment. Our results not only verify the roles of key metabolites in resisting salt stress, but also further evidence that flavone and flavonol biosynthesis and plant growth regulator relate to salt tolerance.

## 1 Introduction

Soil salinization is a major environmental stressor hindering global crop production, risking food security and weakening global efforts towards the Sustainable Development Goals (Wang et al., 2022). Because saline soil affects seed germination and seedling establishment (Farooq et al., 2015), priming crop seeds before germination has emerged as a promising approach to reduce seedling salt stress and enhance crop yields on salinized land (Hussain et al., 2023).

Seed priming involves exposing crop seeds to natural and/or synthetic compounds (Hernandez-Apaolaza, 2022) that cause the activation of early germination events. This makes primed seeds more resilient to environmental stressors, leading to increased survival rates. Seed priming methods include invasive (hydro-, osmo-, halo-, solid matrix, bio- or nano-priming) and non-invasive (magneto, UV-irradiation, γ-radiation, cold plasma, electron and laser priming) processes (Hernandez-Apaolaza, 2022). Among these, hydropriming offers a sustainable and low-cost means to improve seed germination and seedling emergence in salt affect soil (Yan, 2016; Yaghoubian et al., 2022).

Maize (Zea mays) is a globally critical cereal crop that is sensitive to salinized soil conditions (Farooq et al., 2015). Several studies have focused on the effectiveness of priming maize seeds to mitigate salt stress, highlighting the physical-biochemical response in its germination and establishment (Chattha et al., 2022; Hamna et al., 2022; Samra et al., 2022). However, little attention has been paid so far to the metabolic response of hydroprimed maize under salt stress in the early development stage. A systematic assessment of endogenous plant metabolites (metabolomics) can help unravel metabolic networks and shed light on interactions between plants and the environment (Abideen et al., 2022). Roots are in close contact with the soil solution, they are first to confront excessive salinity and are the first places of “line of defence”. Therefore, differences among roots may (partially) underlie distinguishing salt tolerances(Rewald et al., 2013)

In this study, we used a metabolomics approach to study the effect of salt stress of hydroprimed maize to identify the types and variation of differentially accumulated metabolites in roots with the objective to provide a scientific basis for enhancing salt tolerant traits.

## 2 Materials and methods

### 2.1 Hydropriming

Maize seeds (cv Tiantai 316, a high-quality and high-yielding maize variety) were surface sterilized and soaked in deionized water under dark conditions (24 hours; 20 °C). After soaking, the seeds were rinsed (×3) with deionized water and then air-dried (48 hours; room temperature) to back their initial moisture content.

### 2.2 Germination tests

Germination tests (3 replicates; 20 seeds per replicate) involved placing seeds in transparent germination boxes on filter paper wetted with saline water (15 mL; 150 mM NaCl), or deionized water (15 mL) as a control. The seeds were allowed to germinate under light/dark cycles (12/12 hour) at 25 °C for 7 days. Germination was considered to occur when the radicle protruded through the seed coat, with the number germinated seeds recorded daily. The germination potential was calculated as *∑(Gn/Tn)* where *Gn* is the number of germinated seeds on day *n* and *Tn* is day *n*.

Then, the seedling were divided into two parts. One part was oven dried (80°C; 24 hours) and the seedling, root and shoot dry weight recorded. The roots of the other part were immediately used for biochemical and metabolite analyses.

### 2.3 Biochemical analyses

#### 2.3.1 Hydrogen peroxide analysis

Peroxide (H_2_O_2_) levels were determined according to Sergiev et al. (1997). In brief, root sample (0.5 g fresh weight) was homogenized in trichloroacetic acid (5 mL; 0.1% w/v) and centrifuged (12,000 rpm; 15 min). The supernatant (0.5 mL) was mixed with potassium phosphate (0.5 ml; 10 mM; pH7.0) and potassium iodide (1 mL; 1 M) before being allowed to develop for 1 hour in dark conditions. The H_2_O_2_ concentration was then measured spectrophotometrically at 390 nm.

#### 2.3.2 Antioxidant enzymes, lipid peroxidation, and soluble protein analysis

Root samples were first treated with liquid nitrogen and mixed with potassium phosphate buffer (50 mM; pH 7.0) containing Na-EDTA (2 mM) and then centrifuged. Superxoide dismutase (SOD) activity was then measured in the supernatant by its ability to inhibit the reduction of nitroblue tetrazolium at 560 nm (Dhindsa et al., 1981). Peroxidase (POD) activity was assayed according to Chance and Maehly (1955). CAT activity was measured as described by Aebi (1984). Malondialdehyde (MDA) was determined according to the thiobarbituric acid assay described by Heath and Packer (1968). Soluble proteins were measured according to Bradford (1976).

#### 2.3.3 Soluble sugars and proline analysis

Soluble sugars were measured using the anthrone method (Irigoyen et al., 1992) and proline was determined according to Bates et al. (1973).

#### 2.3.4 Sodium and potassium analysis

Roots samples were washed with deionized water followed by air and oven drying(24 hours; 80°C). Dried powder samples (0.1 g) were treated with concentrated nitric acid (10 mL) for 12 h. Each digested material was then diluted with deionized water to a definite volume (100 mL). The contents of Na^+^ and K^+^ were determined by flame photometry according to the method described by Williams and Twine (1960).

### 2.4 Metabolite analyses

#### 2.4.1 Metabolite extraction

Frozen root samples (3 replicates; ~50 mg per replicate) were transferred to Eppendorf tubes (2 mL). Then, methanol/acetonitrile/water (1 mL; 2:2:1 v/v/v) containing ribitol (20 µL; 1 mg/mL) was added as an internal standard. The samples were vortexed (30 s), homogenized using a ground powder system (45 Hz; 10 min), subjected to supersonic processing in ice water (10 min), cultured (-20 °C; 1 hour) and centrifuged (12,000 rpm; 15 min; 4 °C). Then the supernatant (500 µL) was transferred to an Eppendorf tube and dried in a vapor concentrator. The samples were dissolved in acetonitrile/water (160 µL; 1:1 v/v), vortexed (30 s), sonicated (10 min) and centrifuged (12,000 rpm; 15 min; 4 °C). The supernatant (120 µL) was dispensed into glass vials and 10 µL of each sample was mixed into a QC sample for assay analysis.

#### 2.4.2 LC-MS/MS analysis

A Waters Acquity I-Class PLUS ultra-high performance liquid tandem Waters Xevo G2-XS QTOF high-resolution mass spectrometer installed with a Waters Acquity UPLC HSS T3 (1.8µm 2.1*100mm) column was used for metabolomics analyses. The following parameters were applied:

Positive ion mode: mobile phase A: 0.1% formic acid aqueous solution; mobile phase B: 0.1% formic acid acetonitrile. Negative ion mode: mobile phase A: 0.1% formic acid aqueous solution; mobile phase B: 0.1% formic acid acetonitrile. The step elution program was as follows: 0 min, 98% A; 0.25 min, 98% A; 10 min, 2% A; 13 min, 2% A; 13.1 min, 98% A; 15 min, 98% A; the injection volume was 1 µL. MS/MS profiles were obtained using triple TOF-MS based on information acquisition technique (IDA). MS databases were continuously collected and evaluated by acquisition software with full scan survey (Analyst TF 1.7, AB Sciex), and MS/MS spectra were acquired depending on preset parameters. Within each cycle, precursor ions with strengths above 100 were picked and then fragmented at 30 V collision energy (CE) (15 MS/MS events every 50 ms). The electrospray ionization (ESI) source was set to 60 PSI nebulizer pressure, 60 PSI auxiliary gas pressure, 30 PSI air curtain pressure, 650 °C source temperature, and ion spray voltage float (ISVF) of 5000 or -4000 V in positive and negative patterns, respectively.

Primary and secondary mass spectrometry data were collected in MSe mode (MassLynx V4.2, Waters). In each data acquisition cycle, dual-channel data acquisition was performed on both low collision energy and high collision energy at the same time. The low collision energy was 2V, the high collision energy range was 10~40V, and the scanning frequency was 0.2 seconds for a mass spectrum. The parameters of the ESI ion source are as follows: Capillary voltage: 2000V (positive ion mode) or -1500V (negative ion mode); cone voltage: 30V; ion source temperature: 150°C; desolvent gas temperature 500°C; backflush gas flow rate: 50L/h; Desolventizing gas flow rate: 800L/h.

#### 2.4.3 Data analysis

Firstly, peak areas were normalized to the total peak area. Then, principal component analysis and Spearman correlation analysis were used to assess the repeatability of the samples. Identified compounds were searched for classification and pathway information in KEGG, HMDB and lipidmaps databases. T-tests were performed to determine significant differences between each compound. R (programming language) with the ropls software package was used to perform principal component analysis (PCA) and OPLS-DA modeling, with 200 permutations performed to verify the reliability of the model. VIP values were calculated using multiple cross-validation. The difference multiple, P value and the VIP value of the OPLS-DA model was used to screen the differential metabolites (criteria = FC > P<0.05 and VIP > 1). Difference of metabolites of KEGG pathway enrichment significance were calculated using a hypergeometric distribution test.

### 2.5 Validation of metabonomics data by quantitative real-time reverse transcription polymerase chain reaction

To validate our data, four genes (*delta-1-pyrroline-5-carboxylate synthase 2* (*P5CS*, Gene ID:100280719), *caffeic acid 3-O-methyltransferase* (*COMT*, Gene ID:100125646), *catalase isozyme 2*(*CAT2*, Gene ID:542230) and *steroid reductase DET2*(Gene ID:100283443) were selected for qRT-PCR assay, with three biological replicates used for each analysis. The primer used for one internal reference gene (*Actin*) and the four selected genes are shown in **Table S1**. Total RNA was extracted from the maize roots samples treated with either 150 mMNaCl or water by the RNA extraction kit (Monad Biotech, Suzhou, China) following the manufacturer’s instructions. The concentration and integrity of each RNA samples was examined by BioPhotometer measurement and 1% agarose gel. Based on the recommendation of the TUREscript 1st Stand cDNA SYNTHESIS Kit (Aidlab, Beijing, China), 2 µg total RNA was measured to perform the cDNA synthesis. The PCR reactions were performed on the analytik Jena-qTOWER2.2 Real-Time QPCR System using the following program: denaturation at 95°C for 3min followed by 39 cycles of 95 °C for 10 s and 60°C for 30 s, and terminated at 72°C for 60 s. Dissociation curve analysis was performed to determine the target specificity. The 2^-ΔΔCT^ method was used to calculation of the relative expression of selected genes normalized to a maize *Actin* gene.

### 2.6 Statistical analysis

A statistical analysis was carried out using SPSS 19.0 software (IBM). Germination percentage data were arcsine transformed before an analysis of variance. Mean comparisons were performed using a Duncan test (5% probability).

## 3 Results

### 3.1 Effect of salt stress on germination and seedling traits

**Figure 1:**
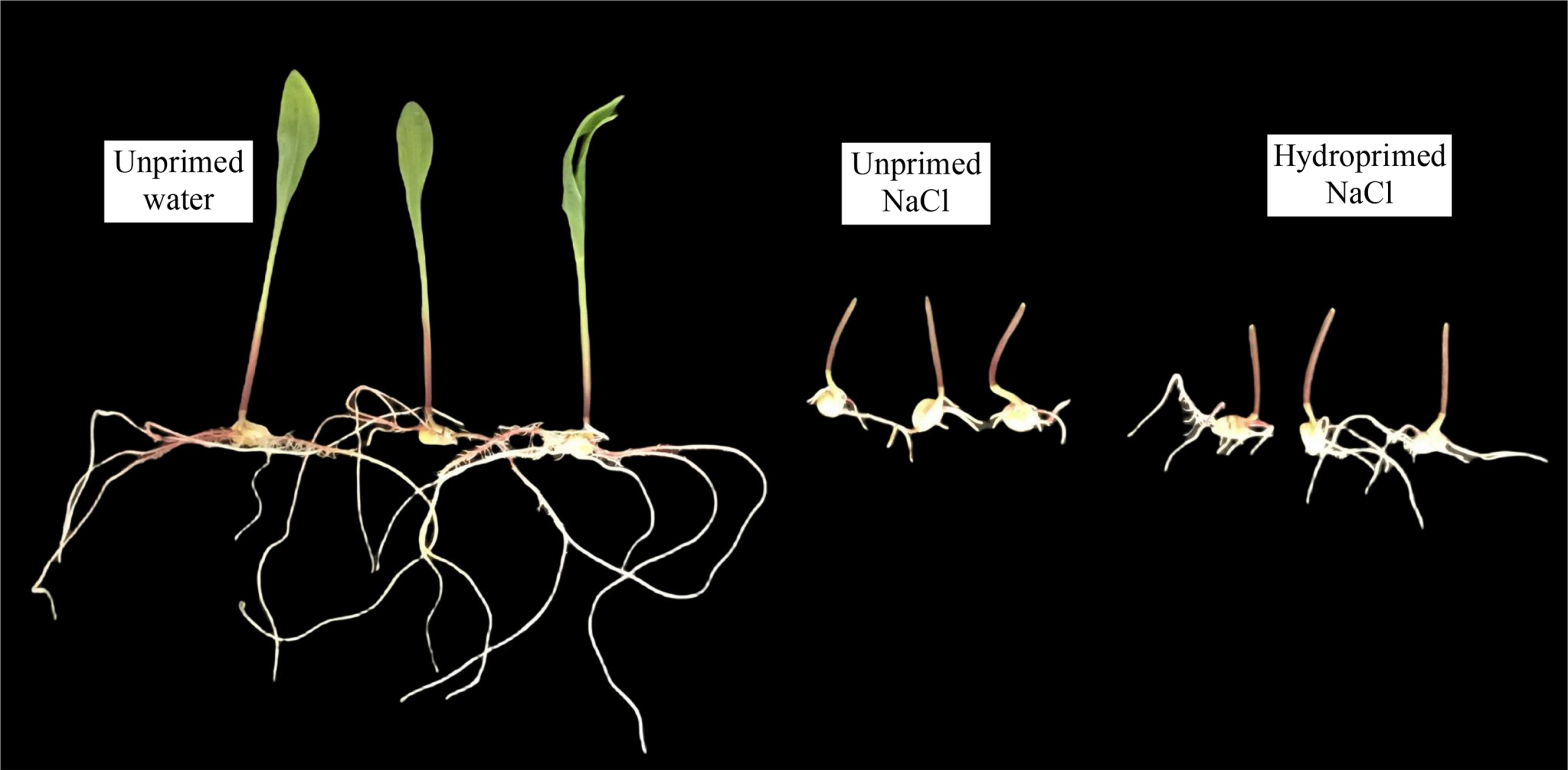
Comparative growth of seedlings of unprimed and hydroprimed samples under salt stress (NaCl) and control (unprimed water) on 7^th^ day.

**Figure 2:**
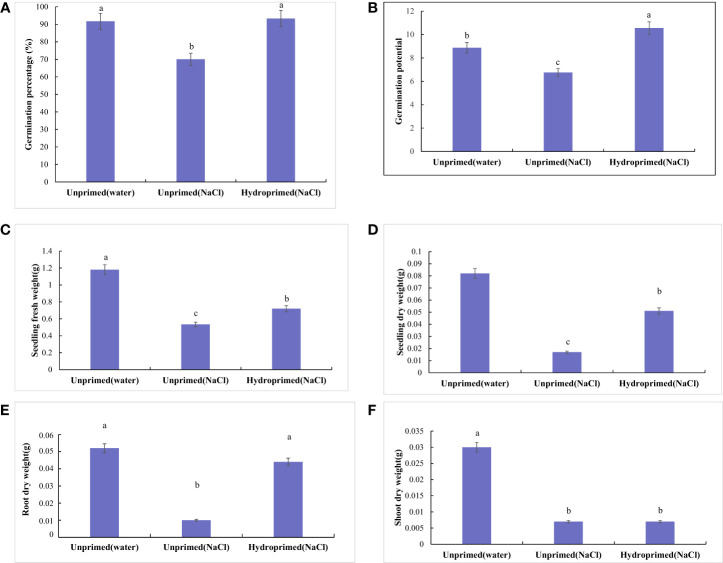
Influence of salt stress and hydropriming treatment on germination [Germination percentage **(A)** and Germination potential **(B)**] and seedling traits [Seedling fresh weight **(C)**, Seedling dry weight **(D)**, Root dry weight **(E)** and Shoot dry weight **(F)**] of maize. Different letters (a and b) indicate significant differences among treatments according to Duncan’s test (*p* < 0.05). Error bars indicate ± SE of mean (n = 3).

The effect of salt stress on germination and seedling traits is illustrated in **Figures 1**, **2**; **Figure S1**. Salt stress severely inhibited the germination of unprimed samples and significantly decreased the germination traits [germination percentage (**Figure 2A**) and germination potential (**Figure 2B**)] and early seedling growth [seedling fresh weight (**Figure 2C**), seedling dry weight (**Figure 2D**), root dry weight (**Figure 2E**) and shoot dry weight (**Figure 2F**)] compared to the control. In comparison, hydroprimed sample displayed significantly improved germination and early seedling growth in all aspects apart from shoot dry weight. The germination percentage (**Figure 2A**) and root dry weight (**Figure 2E**) of hydroprimed samples achieved level not significantly different to the control (i.e., unhindered by salt conditions). Furthermore, the germination potential of primed samples actually superseded that of the control (**Figure 2B**). However, the shoot dry weight of primed samples was not significantly different to unprimed samples under salt stress (**Figure 2F**).

### 3.2 Effect of salt stress and hydropriming on physiological and biochemical traits

**Figure 3:**
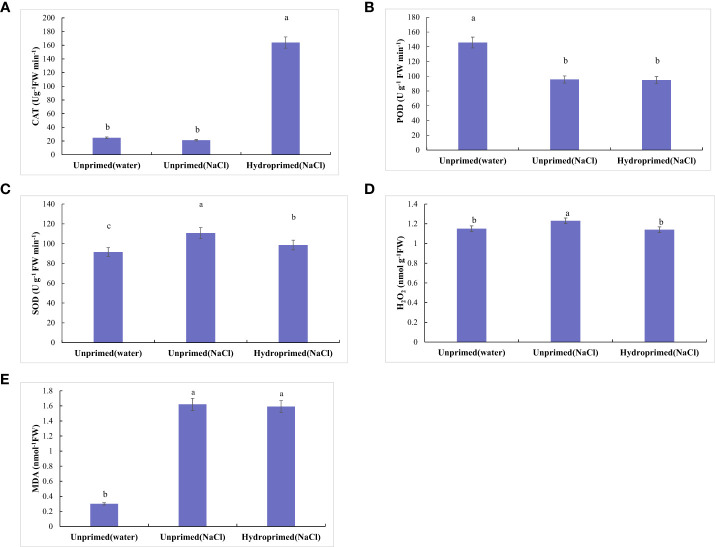
Influence of salt stress and hydropriming treatment on the activities of CAT **(A)**, POD **(B)**, SOD **(C)**, and content of H_2_O_2_ **(D)**, MDA **(E)** in maize roots. Different letters (a and b) indicate significant differences among treatments according to Duncan’s test (*p* < 0.05). Error bars indicate ± SE of mean (n = 3).

**Figure 4:**
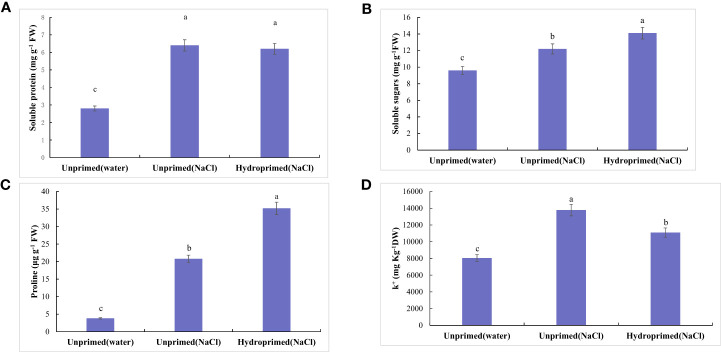
Influence of salt stress and hydropriming treatment on soluble protein **(A)**, soluble sugars **(B)**, proline **(C)**, K^+^/Na^+^ ration **(D)** in maize roots. Different letters (a and b) indicate significant differences among treatments according to Duncan’s test (*p* < 0.05). Error bars indicate ± SE of mean (n = 3).

The effect of salt stress on the physiological and chemical traits of unprimed and hydroprimed samples is shown in **Figures 3**, **4**. We found that salt stress had no significant effect on CAT activity of the unprimed samples, while hydroprimed samples had significantly increased CAT activity under salt stress (**Figure 3A**). Salt stress decreased POD activity of unprimed samples under salt stress, while hydropriming treatment had no significant effect on POD activity under salt stress (**Figure 3B**). For unprimed samples, SOD activity (**Figure 3C**), H_2_O_2_ (**Figure 3D**), MDA (**Figure 3E**) levels all significantly increased under salt stress. SOD activity of hydroprimed samples was lower than the unprimed one and significantly higher than the control (i.e., not salt stress effect)(**Figure 3C**). The H_2_O_2_ content of hydroprimed samples was lower than the unprimed one and not significantly different to the control (i.e., not salt stress effect) (**Figure 3D**). MDA content of hydroprimed samples was not significantly different to the unprimed samples (**Figure 3E**).

Soluble protein (**Figure 4A**), soluble sugars (**Figure 4B**), proline(**Figure 4C**) levels all significantly increased under salt stress, hydropriming treatment had no significant effect on soluble protein under salt stress (**Figure 4A**). Soluble sugars (**Figure 4B**) and proline (**Figure 4C**) levels in primed samples increased. Salt stress decreased K^+^/Na^+^ ration of unprimed samples under salt stress, while hydropriming treatment had no significant effect on K^+^/Na^+^ ratio under salt stress (**Figure 4D**).

### 3.3 Change in metabolites and metabolic pathways in unprimed samples in response to salt stress

**Figure 5:**
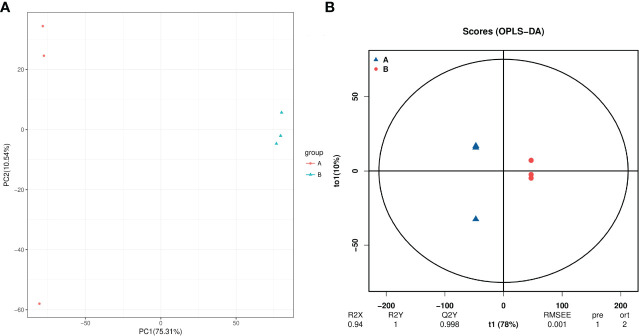
PCA **(A)** and PLS-DA **(B)** analyses of metabolic profiles of roots from unprimed maize seeds under with and without salt stress. group A, roots from unprimed maize seeds without salt stress; group B, roots from unprimed maize seeds under salt stress.

A total of 3418 intracellular metabolites were detected(**Table S2**), for which we compared the differences between the roots of unprimed samples with and without salt stress by a multivariate statistical analysis. The PCA score plot shows two principal components accounting for 86.1% of the variability (**Figure 5A**), while the OPLS-DA score plot (R^2 =^ 0.94; Q^2 =^ 0.998) shows a clear distinction in the metabolite profiles between the salt-stressed roots and the control (**Figure 5B**).

We screened 2322 differential metabolites between the roots from unprimed seeds under control and salt stress condition (VIP > 1; *p* < 0.05) (**Table S3**). Among them, 1278 metabolites significantly increased and 1044 metabolites significantly decreased in the NaCl-stressed samples compared to the control (**Table S3**). VIP scores obtained from PLS-DA and OPLS-DA analyses indicated that some metabolites made important contribution to the separation of the salt stresses samples from control. The 10 most important metabolites identified from VIP and PCA scores were in the order of: Ethisterone (progesterone); Menadione (Vitamin K3); 10-Deacetyl-2-debenzoylbaccatin III; Neomycin B; Pelargonidin 3-O-(6-caffeoyl-beta-D-glucoside); 5-O-beta-D-glucoside; 6-Gingerol; Dexamethasone, N6-(L-1,3-Dicarboxypropyl)-L-lysine; Tacrolimus and Quinate (**Table S3**).

**Figure 6:**
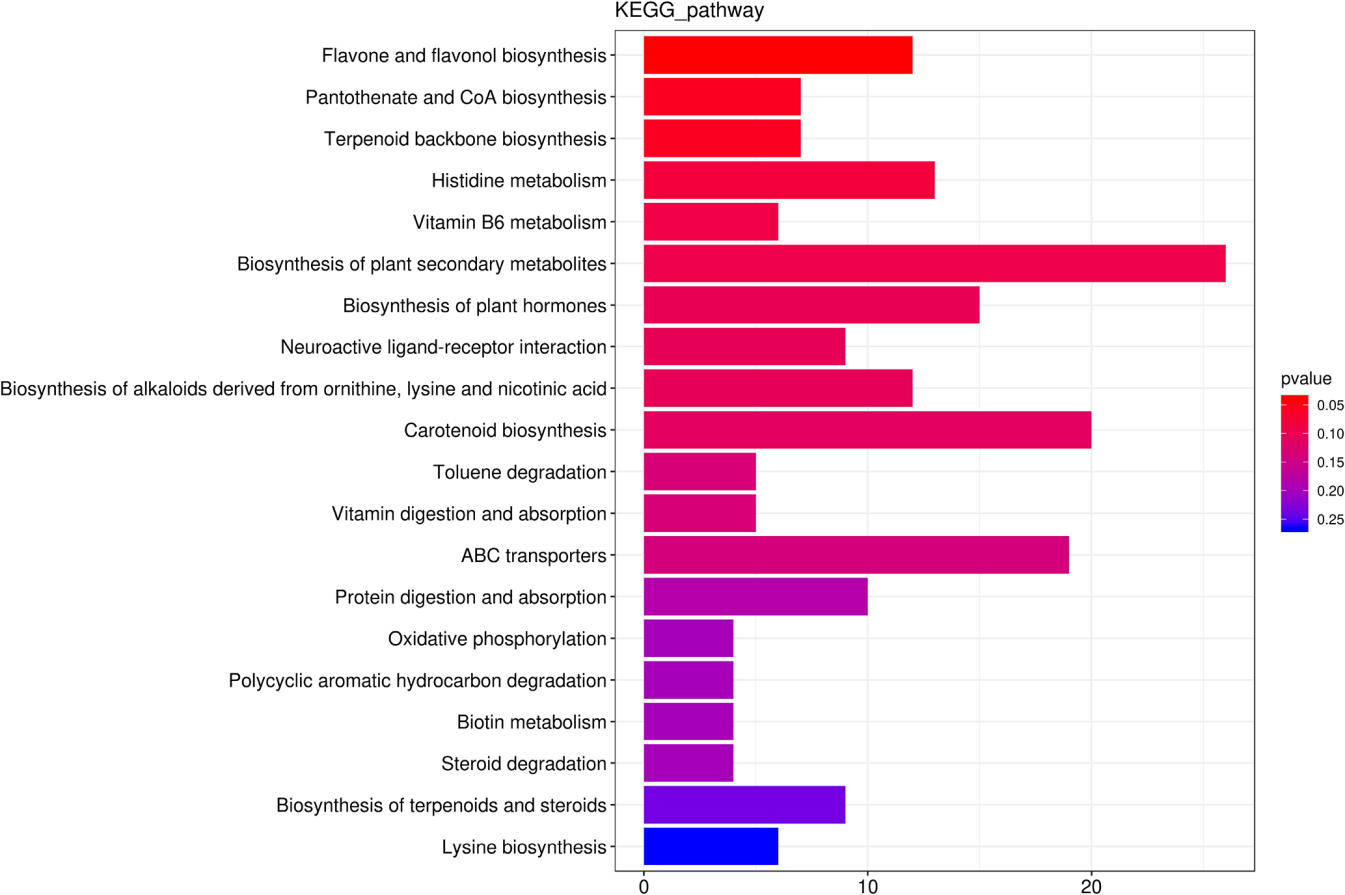
The top 20 the Kyoto Encyclopedia of Genes and Genomes (KEGG)-enriched metabolism pathway of differential metabolites between roots from unprimed maize seeds under with and without salt stress.

We identified 256 metabolic pathways with 421 differential metabolites. Among these pathways, Flavone and flavonol biosynthesis, CoA biosynthesis, Vitamin B6 metabolism, Terpenoid backbone biosynthesis and Histidine metabolism were the top five predominant metabolic pathway (**Figure 6**).

Flavone and flavonol biosynthesis was the only pathway that was significantly (p<0.05) affected by salt stress with an increase for 10 metabolites and a decrease for 2 metabolites. The concentration of Luteolin 7-O-beta-D-glucoside (prunin), 3,7,4’-Tri-O-methylquercetin, Chrysoeriol, Scolymoside(luteolin 7-O-rutinoside), Kaempferol 3-O-glucoside, 3,7-Di-O-methylquercetin, Luteolin 7-O-glucuronide, Quercetin 3-methyl ether, Rutin, Quercetin 3-O-glucoside increased 18.44-fold, 18.38-fold, 11.37-fold, 4.36-fold, 3.73-fold, 3.60-fold, 3.20-fold, 3.06-fold, 2.30-fold, 2.06-fold, respectively. Kaempferol and Acacetin decreased.

### 3.4 Change in metabolites and metabolic pathways in hydroprimed samples in response to salt stress

**Figure 7:**
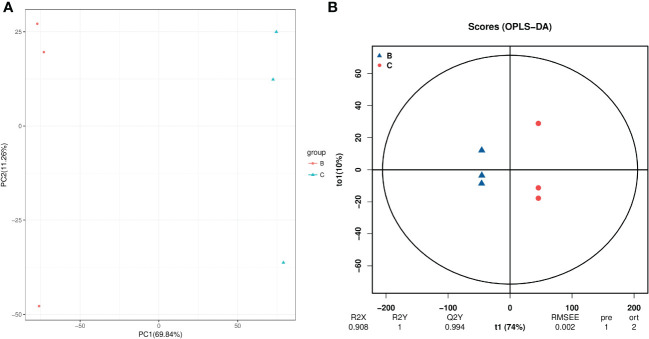
PCA **(A)** and PLS-DA **(B)** analyses of metabolic profiles of roots from unprimed and hydroprimed maize seeds under salt stress. group B, roots from unprimed maize seeds under salt stress; group C, roots from hydroprimed maize seeds under salt stress.

For hydroprimed samples, the results of the PCA score plot showed that the samples were clearly separated by two principal components, which explained 82.4% of the variability (**Figure 7A**). The OPLS-DA score plot (R^2 =^ 0.908; Q^2 =^ 0.994) also shows a clear distinction in the metabolite profile of unprimed and hydroprimed samples under salt stress (**Figure 7B**).

A total of 2186 differential metabolites were screened (VIP > 1; *p* < 0.05), between the roots from unprimed and hydroprimed seeds under salt stress condition (**Table S4**). Among them, 873 metabolites significantly increased and 1313 metabolites significantly decreased in the hydroprimed samples compared to the control (**Table S4**). VIP scores obtained from PLS-DA analysis and loading plots from OPLS-DA (Figure not presented) analysis indicated that 5-Methyltetrahydrofolate and some metabolites were of important contributing to the separation of the hydropriming treatment samples from control. The top 10 most important contributory obtained from VIP and PCA scores were 5-Methyltetrahydrofolate, 24-Hydroxy-beta-amyrin, Dethiobiotin, 3alpha,7alpha-Dihydroxy-5beta-cholestane, Piperideine, (S)-Autumnaline, Formononetin 7-O-glucoside-6’’-O-malonate, 2-Heptyl-4(1H)-quinolone, 2-Hydroxycinnamic acid, Lupinine, 2-Methylglutaric acid (**Table S4**).

**Figure 8:**
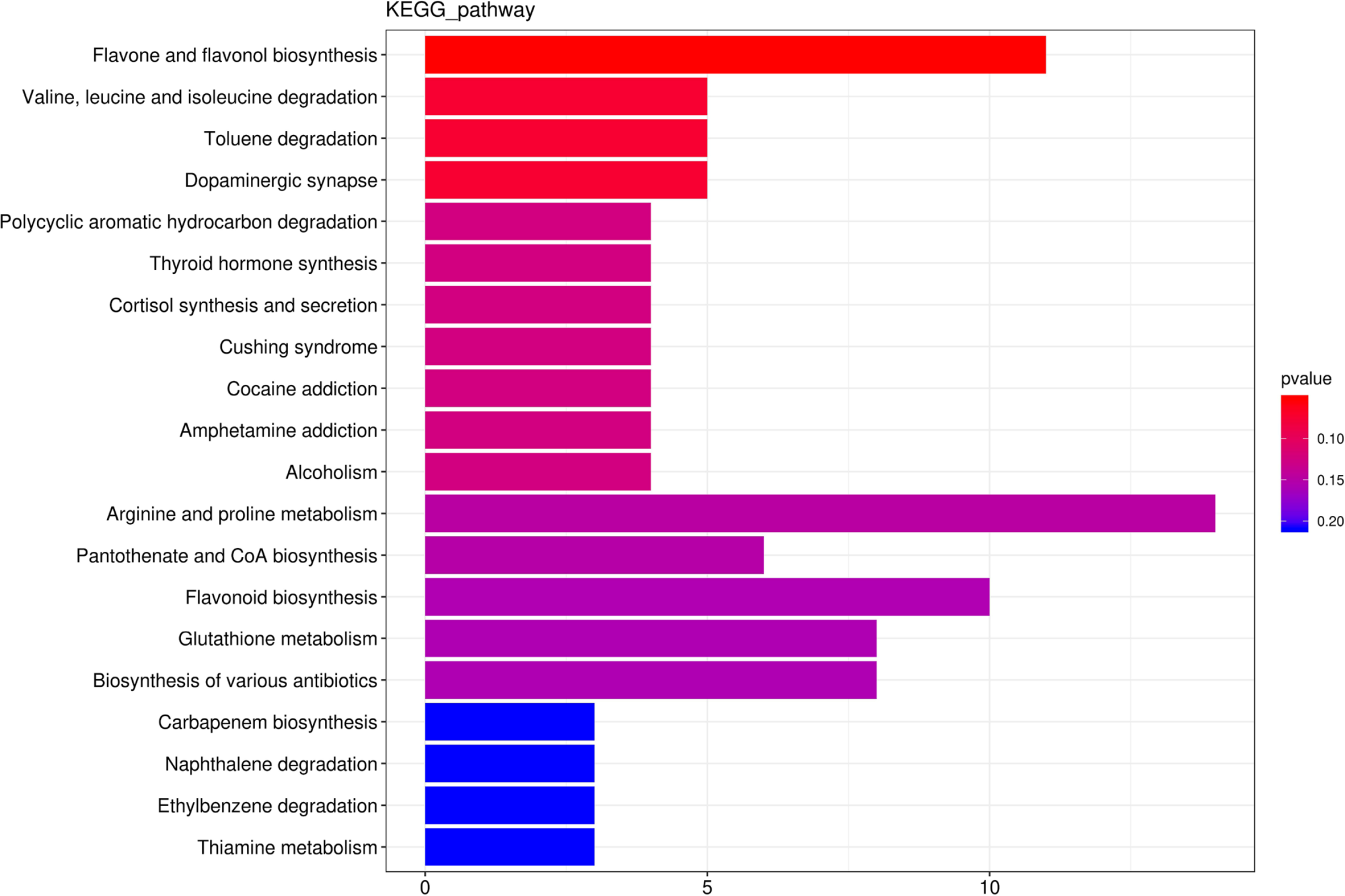
The top 20 the Kyoto Encyclopedia of Genes and Genomes (KEGG)-enriched metabolism pathway of differential metabolites between roots from unprimed and hydroprimed maize seeds under salt stress.

Among the 2186 differential metabolites, 374 differential metabolites involved in 254 metabolic pathways. Flavone and flavonol biosynthesis, Valine,leucine and isoleucine degradation, Toluene degradation, Dopaminergic synapse, Polycyclic aromatic hydrocarbon degradation were the top five predominant metabolic pathway (**Figure 8**). Thus, none of these metabolic pathways was significantly affected by hydropriming treatment.

### 3.5 Shared features of salt stress and hydropriming treatment on metabolites

**Figure 9:**
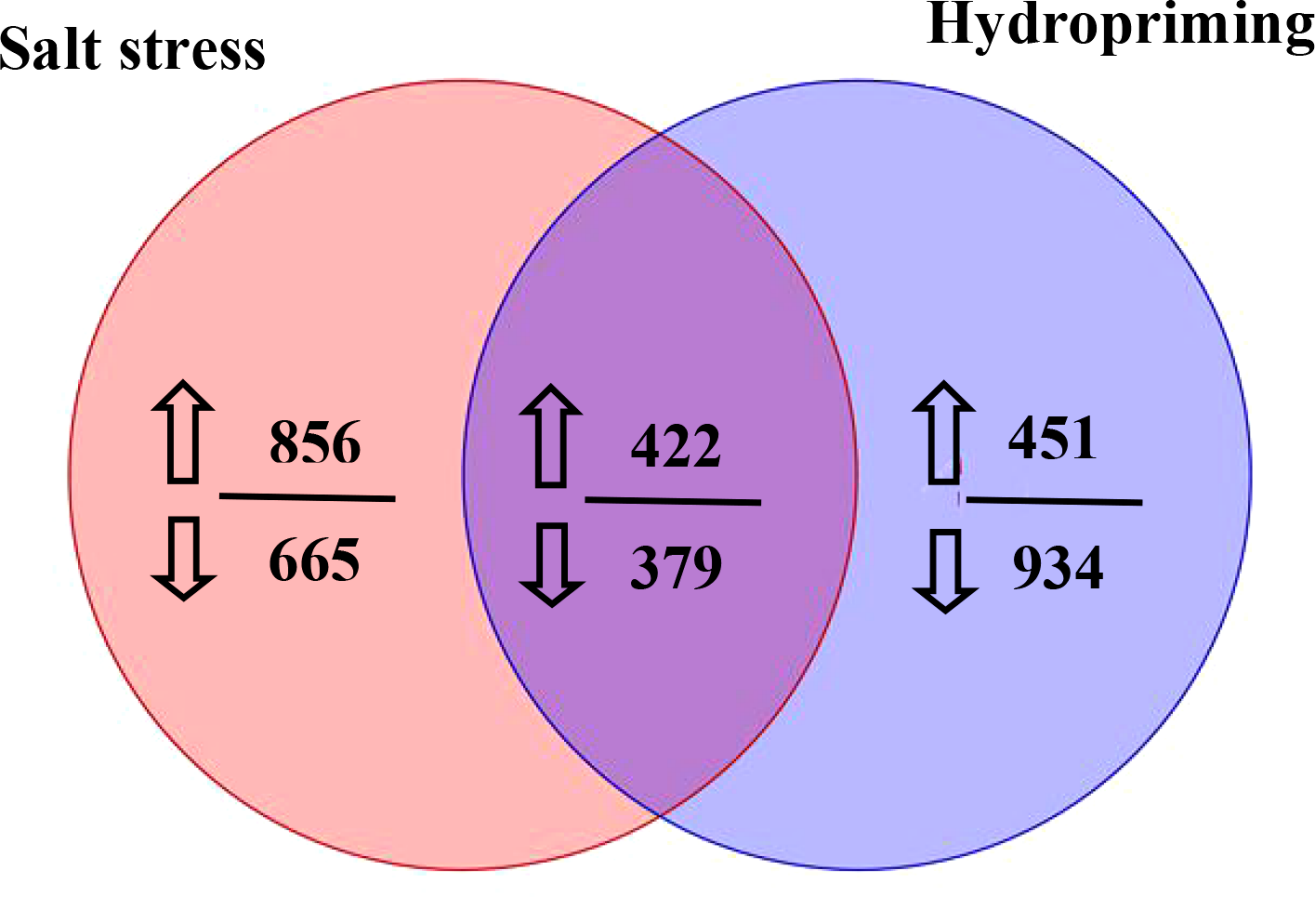
Venn diagram showing the number of metabolites that increased or decreased in the salt stressed and hydroprimed seedlings. The numbers next to up and down arrows indicated the number of upregulated and downrugulated differentially metabolites.

The metabolites between salt stress and hydropriming treatment maize roots were compared in this study. Between the salt stress and hydropriming treatment group, 422 increased (**Table S5**) and 379 decreasd (**Table S6**) overlapping metabolites were identified (**Figure 9**). We noticed that plant growth regulator, such as melatonin, gibberellin A8 and estrone increased, while abscisic acid and brassinolide decreased in both treatment.

### 3.6 Expression of selected genes

**Figure 10:**
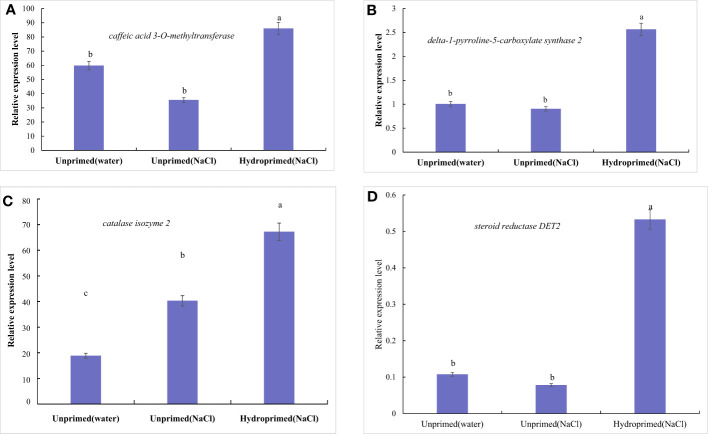
Influence of salt stress and hydropriming treatment on relative expression levels of *delta-1-pyrroline-5-carboxylate synthase 2* **(A)**, *caffeic acid 3-O-methyltransferase* **(B)**, *catalase isozyme 2* **(C)** and *Steroid reductase DET2* **(D)** by qRT-PCR. Different letters (a and b) indicate significant differences among treatments according to Duncan’s test (*p* < 0.05). Error bars indicate ± SE of mean (n = 3).

The effects of salt stress on the expression of four genes of unprimed and hydroprimed samples is shown in **Figures S2** and **10**. We found that salt stress had no significant effect on the expression of *P5CS, COMT* and *steroid reductase DET2* in the unprimed samples (**Figures 10A, B, D**), and increased the expression of *CAT2* (**Figure 10C**). Hydroprimed samples had significantly increased the expression of four genes under salt stress(**Figures 10A–D**).

## 4 Discussion

### 4.1 Effects of salt stress and hydropriming treatment on germination and early seedling growth

Exogenous and endogenous factors including salinity affect maize germination and seedling growth (Mahara et al., 2022). The present study indicated that 150 mmol.L^-1^ NaCl significantly inhibited germination and seedling growth. The germination traits (germination percentage and germination potential) and seedling traits (seedling dry weight, root dry weight and shoot dry weight) of unprimed seeds significantly decreased under salt stress. We found that hydropriming treatment significantly improved germination and seedling growth of maize under salt stress, which is in agreement with previous research (Mahara et al., 2022). The dry weight of seedlings and shoots of hydroprimed samples were higher than those of seedlings from unprimed seeds under salt stress, but lower than those of seedlings from unprimed seeds. Thus, the dry weight of roots from hydroprimed seeds reach the level of that of unprimed seeds under non-salt stress conditions, which means that the improvement in seedling growth mainly manifested in promoting root growth.

### 4.2 Physiology and biochemical responding to salt stress and hydropriming

Previous studies have indicated that salt stress often leads to excessive production of reactive oxygen species (ROS), such as superoxide (O_2_^•-^), hydroxyl radical (^•^OH) and hydrogen peroxide (H_2_O_2_), which attack biomacromolecules and results in lipid peroxidation, protein degradation and membrane damage (Choudhary et al., 2020). The antioxidant enzymes SOD, CAT and POD are crucial to alleviating oxidative damage by scavenging ROS (Choudhary et al., 2020). SOD is the first line of antioxidant defense and transforms superoxide radicals to H_2_O_2_ and form hydroxyl radicals (Choudhary et al., 2020; Hai et al., 2022). In this study, we found that SOD activity significantly increased under salt stress. MDA is an index of lipid peroxidation and accumulates when the antioxidant defenses fail to maintain ROS levels (Choudhary et al., 2020). We found that MDA levels increased in salt stressed roots, attributed to excessive H_2_O_2_. Importantly, hydropriming significantly decreased H_2_O_2_ levels under salt stress and curtailed an increase MDA. The positive effect of hydropriming is likely related to increased antioxidant CAT activity, which detoxifies H_2_O_2_ to oxygen and water (Choudhary et al., 2020). However, due to the decrease of SOD activity, MDA content of primed samples was significantly higher than unprimed samples under salt stress.

In present study, the expression of *CAT2* increased while catalase activity remained unchanged in salt stress roots, which may be caused by the decreased expression of other isoenzymes of catalase. In hydropeimed roots, increased CAT activity appeared linked to the increase expression of *CAT2*.

The influence of salt stress on soluble protein content generally relates to salt tolerance of plants (Gandonou et al., 2011). Previous studies of sugarcane plants have indicated that salt stress leads to an increase in soluble proteins in salt tolerant cultivars and a decrease in salt sensitive cultivars (Gandonou et al., 2011). The increase is probably related to stress proteins, which play important roles in osmotic adjustment under stress conditions (Timperio et al., 2008). The present findings elucidated that salt treatment significantly increased soluble protein content in maize seedlings under salt stress. Hydropriming did not increase further the amount of soluble protein produced under salt stress.

Plants also accumulate proline and sugars under water stress (Boughalleb et al., 2020). Soluble sugars are involved in osmo-regulation in plants exposed to osmotic stress (Boughalleb et al., 2020). Proline has been considered a compatible osmoregulator, contributing to cellular osmotic adjustment and cellular and physiological homeostasis maintenance in salt-stressed plants (Boughalleb et al., 2020). The present findings indicated that salt stress in unprimed and hydroprimed samples significantly augmented proline and sugar content. This is in agreement with previous data showing enhanced proline and sugar content contributing to mitigating adverse effects of salt stress (Boughalleb et al., 2020).

*Delta-1-pyrroline-5-carboxylate synthase 2* (*P5CS*) encoding delta1 -pyrroline-5-carboxylate synthase (P5CS) has been reported as the main responsible gene in proline biosynthesis (Lutts et al., 1999). In present study, the interesting point is the differences between high proline content and low expression levels of *ZmP5CS2* in salt stressed roots, which indicated that the accumulation of proline can not be only a result of increased stress-induced expression of *ZmP5CS2.* In the meantime, the accumulation of proline accompanying with the increase expression of *ZmP5CS2* in hydroprimed roots showed *ZmP5CS2* seem to relate to salt tolerance.

K^+^/Na^+^ ratio is often used as a salt tolerance criterion and higher K^+^/Na^+^ ration accompanied with better growth under salt stress (Chen et al., 2023). Present study indicated the K^+^/Na^+^ ratio decreased under salt stress, while hydropriming treatment had no significant effect on K^+^/Na^+^ under salt stress. This suggests that the enhanced salt tolerance of hydropriming treatment was not caused by ion homeostasis.

### 4.3 Metabolites and metabolic pathway responding to salt stress

We found that Ethisterone (progesterone) was the most important metabolite produced in the roots grown from unprimed seeds in response to salt stress. Progesterone is a steroid hormone and ubiquitous in plants at low levels (Li et al., 2022), which enhances antioxidant enzyme activity and affects the K^+^/Na^+^ ratio and pigment content (Li et al., 2022). The amount of progesterone in the roots of unprimed samples significantly decreased under salt stress, while it significantly increased in hydroprimed samples, in accord with previous results (Li et al., 2022).

Menadione is a superoxide-releasing compound and generates ROS through redox cycling (Loor et al., 2010). The metabolite in the roots of unprimed samples significantly increased in response to salt stress. Hydropriming also significantly increased it. We interpret that the accumulation of ROS resulting from salt stress and hydropriming signals the production of antioxidant enzymes such as catalase to scavenge ROS, which otherwise accumulates to potentially damaging levels. Previous research has shown that exogenous application of menadione inducing a mild oxidative stress, leading to chilling tolerance in maize seedlings (Prasad et al., 1994).

10-deacetyl-2-debenzoylbaccatin III (10-DAB III) is an important precursor for taxol synthesis (Yu et al., 2021). Salt stress tolerance requires microtubule disassembly (Zhou et al., 2017a), however, the stability of microtubule with taxol reduces the survival of seedlings under salt stress (Zhou et al., 2017a). We found that 10-Deacetyl-2-debenzoylbaccatin III significantly decreased under salt stress. The decrease in 10-Deacetyl-2-debenzoylbaccatin III may result in the decrease of taxol (not detected) and lead to the depolymerization of the cortical microtubule, which increased the survival of seedlings under salt stress.

Neomycin B is an aminoglycoside antibiotic, which displaces divalent metal ions bound to RNA and inhibits translation in prokaryotes (Waldsich et al., 1998). We found that Neomycin B significantly decreased due to salt stress and hydropriming. The decreased of Neomycin B results in an increase of translation, which is consistent with the increase of soluble proteins under salt stress and hydropriming.

Pelargonidin 3-O-(6-caffeoyl-beta-D-glucoside) 5-O-beta-D-glucoside is a acylated anthocyanin, stimulated by increasing concentrations of NaCl in seedlings (Eryilmaz, 2006).It has been shown to confer significant salt stress tolerance in *Brassica napus* L (Kim et al., 2017). We found that salt stress and hydropriming treatment both significantly increased the content of Pelargonidin 3-O-(6-caffeoyl-beta-D-glucoside) 5-O-beta-D-glucoside.

[6]-gingerol possess strong antioxidant properties, decreases peroxidation of phospholipid liposomes in the presence of iron(III) and ascorbate (Aeschbach et al., 1994). We found a decrease of [6]-Gingerol accompanying the increase of H_2_O_2_ under salt stress. Hydropriming significantly increased [6]-Gingerol with the decrease of H_2_O_2_.

Dexamethasone is a potent glucocorticoid receptor agonist and dexamethasone treatments leading to the activation of mitogen-activated protein kinase3 (MPK3) and MPK6, and depolymerization of the cortical microtubules (Zhou et al., 2017b). Zhou et al. (2017) reported that the activation of MPK3 and MPK6 affects the stability of microtubules, which significantly increased the surviving of seedlings under salt stress (Zhou et al., 2017b). We found that dexamethasone decreased under salt stress for unprimed samples and increased for hydroprimed samples. The variation trend of dexamethasone was consistent with that of seed germination and early seedling growth.

*N*6-(L-1,3-dicarboxypropyl)-L-lysine is a key intermediate in the α-amino adipate pathway for L-lysine biosynthesis (Kiyota et al., 2015), whereas lysine significantly represses proline and pipecolic acid synthesis (Kiyota et al., 2015). We found that *N*6-(l-1,3-dicarboxypropyl)-L-lysine and L-lysine both decreased and proline increased due to salt stress for unprimed and hydroprimed samples.

Tacrolimus is a calcineurin pathway inhibitor (Ma et al., 2005). Calcineurin is a conserved Ca^2+^-calmodulin-dependent serine-threonine-specific protein phosphatase and has multiple functions including regulating ionic homeostasis (Ma et al., 2005). Previous research indicated that the expression of mouse calcineurin protein improved the salt stress tolerance of rice, partly due to limiting Na^+^ accumulation in the roots (Ma et al., 2005). We found a decrease of tacrolimus under salt stress, which likely decreased the inhibition of calcineurin and, thus, increased seedling survival under salt stress.

Quinate is an important marker of salt stress involved in the shikimate pathway in biosynthesis of aromatic amino acid, including tyrosine, phenylalanine and tryptophan (Shelden et al., 2016). We found that quinate significantly increased in the roots of unprimed and hydroprimed samples under salt stress. Tyrosine and phenylalanine both significantly decreased under salt stress. Changes in tryptophan were not obvious. Thus, increased quinate levels may relate to decrease used as a precursor for aromatic amino acid synthesis.

**Figure 11:**
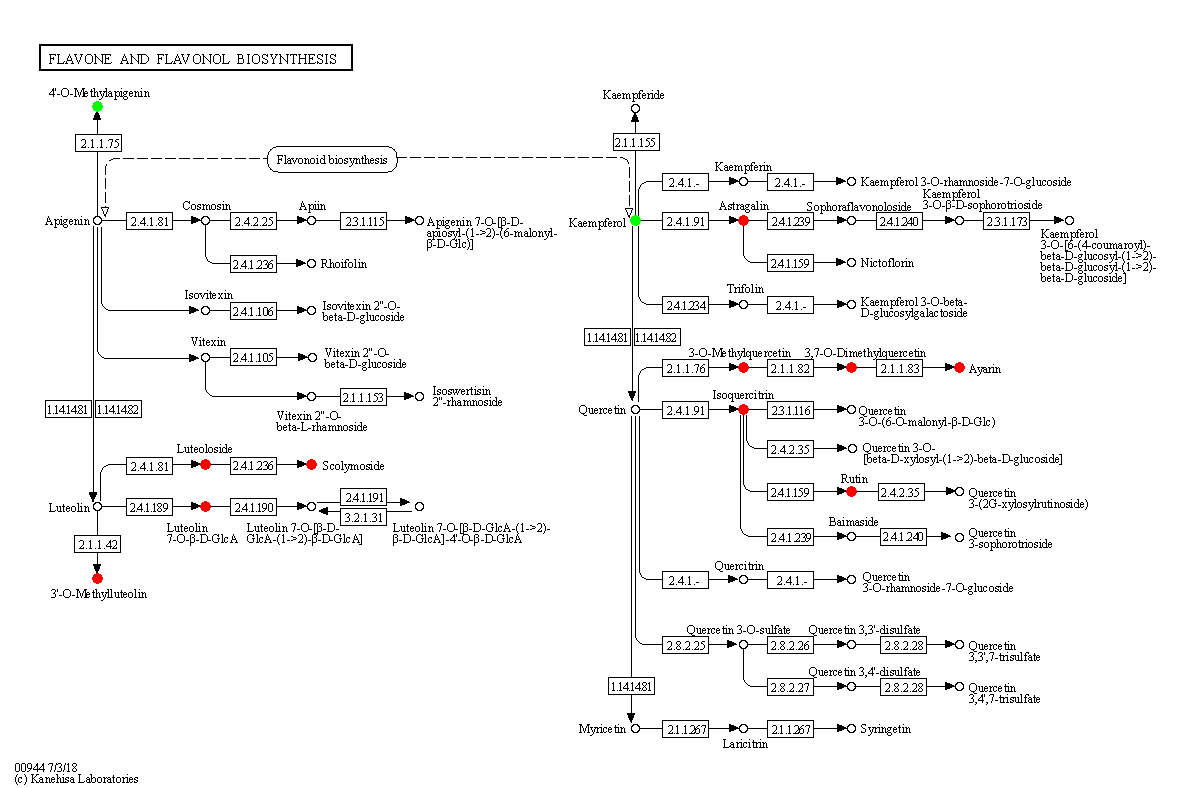
Changes in metabolic pathways of maize roots responding to salt stress. Red-marked metabolites indicated that the metabolite concentration increased significantly under salt stress(p<0.05). Green-marked metabolites indicated that the metabolites concentration decreased significantly under salt stress(p<0.05). Black-marked metabolites showed no significant change.

Salt stress also influences metabolic pathways. We found that flavone and flavonol biosynthesis was the most significantly affected among 256 pathways affected by NaCl stress. Related metabolites, including Luteolin 7-O-beta-D-glucoside, 3,7,4’-Tri-O-methylquercetin, Chrysoeriol, Scolymoside, Kaempferol 3-O-glucoside, 3,7-Di-O-methylquercetin, Luteolin 7-O-glucuronide, Quercetin 3-methyl ether, Rutin, Quercetin 3-O-glucoside were significantly increased under salt stress. In contrast, the content of Acacetin and Kaempferol significantly decreased under salt stress. Among the 12 differential metabolites, 10 metabolites increased and 2 metabolites decreased, suggesting that salt stress activated this pathway in seedlings. Luteolin-7-O-beta-D-glucoside(Luteoloside) exhibited strong scavenging effect on active oxygens and increase under salt stress (Cai et al., 2020). 3,7,4’-Tri-O-methylquercetin was regarded as a contributor to the antioxidant of *Phragmites* under copper stress (Wu et al., 2022). Chrysoeriol possess a potent antioxidant activity and regarded as an important biomarker for salt tolerance in hulless barley (Wang et al., 2019). Scolymoside(luteolin 7-O-rutinoside) exhibited higher antioxidant activity than that of L-ascorbic acid and significantly increased under water-stressed conditions (Kim et al., 2000). Kaempferol 3-O-glucoside possess in vitro antioxidant properties (Taiwo et al., 2019). Griesser et al. (2015) discovered that prolonged drought stress leads to an increase of kaempferol-3-*O*-glucoside. 3,7-Di-O-methylquercetin was found to be related to flavonoids biosynthesis, which involves in plant defense against pathogens, herbivores, and environmental stress (Treutter, 2005). Luteolin-7-O-glucuronide possesses antioxidant activities and may act as reactive oxygen species scavengers, which was associated with the protective role in olive trees under field drought conditions (Araújo et al., 2021). Quercetin 3-methyl ether is the active antioxidant principle in the fruits and stems of Opuntia ficus-indica and markedly inhibited lipid peroxidation and scavenged free radicals (Dok-Go et al., 2003). Rutin scavenged hydroxyl radical and prevented K^+^ leak in quinoa and broad beans under salinity treatment (Ismail et al., 2015). Quercetin 3-O-glucoside exhibits high antioxidant effect and moderate salinity induce the synthesis of quercetin-3-*O*-glucoside (Sgherri et al., 2017). Our results are consistent with previous studies which reported that flavones and flavonol accumulated in plants exposed to salt stress, and that they enhanced plant salinity tolerance *via* scavenging ROS (Zhang et al., 2017). Pathway diagrams are portrayed in **Figure 11**.

### 4.4 Metabolites and metabolic pathway responding to hydropriming under salt stress

We found that 5-Methyltetrahydrofolate was the most important metabolite produced in the roots of hydroprimed samples under salt stress. As a methyl donor, the methyl group of 5-methyltetrahydrofolate was transform to l-homocysteine resulting in the formation of methionine (Xu et al., 2010). Methionine is used in proteins synthesis or converted into *S*-adenosylmethionine. The methyl group of *S*-adenosylmethionine is applied to DNA and RNA modification and the synthesis of plant structural components (Xu et al., 2010). The carbon part of *S*-adenosylmethionine is used to generate ethylene and polyamines (Xu et al., 2010). Ethylene is a pivotal regulator of salt stress tolerance in plants (Riyazuddin et al., 2020). Polyamines can regulate the replication of DNA and cell division and the content of polyamines significantly increased under salt stress (Flores et al., 1989). Our results indicate that hydropriming significantly increased the level of endogenous 5-methyltetrahydrofolate under salt stress, in agreement with previous research (Riyazuddin et al., 2020).

24-Hydroxy-beta-amyrin is the precursor of soyasapogenol B (Tamura et al., 2018). Soyasapogenol B is a plant metabolite belonging to the group of triterpenoid saponins and is reported to stimulate germination of barley seeds (Evidente et al., 2011). We found that hydropriming significantly increased 24-OH-β-amyrin in maize roots under salt stress.

Dethiobiotin is the precursor of biotin (Wienhausen et al., 2022). Biotin is a water-soluble vitamin and required for normal cellular function and growth (McMahon, 2002). Biotin addition can enhance salt tolerance (shown in *Torulopsis mogii*) by accumulating glycerol and trehalose, improving fatty acid synthesis with longer chains and higher saturation, which enhances the stability of the cell structure, increasing PM-ATPase activity, and decreasing the ratio of K^+^/Na^+^(Chen et al., 2015). It is known that the relative proportions of saturated and unsaturated fatty acids affect the fluidity and permeability of the cell membrane (Chiang et al., 2005).

3α, 7α-dihydroxy-5β-cholestane is the intermediate bile acid synthesis (Kimura et al., 1988). In the presence of iron ions, hydrophobic bile acids may enhance lipid peroxidation (Sreejayan and Ritter, 1998). We found that 3α,7α-Dihydroxy-5β-cholestane increased in roots of unprimed and hydroprimed samples under salt stress, with no bile acid was detected, which indicates that the increase of 3α,7α-Dihydroxy-5β-cholestane was due to decreased synthesis of bile acid. A decrease of bile acid may result in a decrease of lipid peroxidation.

(S)-autumnaline is the precursor of cadaverine, which affects growth and development under normal and stress environments (Kuznetsov et al., 2007). Cadaverine decreases salt-induced impact, which is attributed to its antioxidative function scavenging of free radicals. We found that hydropriming significantly increased (S)-autumnaline and colchicine in mazie roots under salt stress, which was in accordance with the observed decrease in H_2_O_2_.

Formononetin 7-O-glucoside-6’’-O-malonate is an intermediate in the elicitor-induced formation of pterocarpan phytoalexins, whose synthesis is known to be induced by biotic and abiotic stress factors (Dixon and Paiva, 1995). We found that hydropriming significantly increased Formononetin 7-O-glucoside-6’’-O-malonate in maize roots under salt stress.

2-heptyl-4(1H)-quinolone is the core structure of several alkaloids and may act as a messenger molecule in a cell-cell-communication pathway (Déziel et al., 2004). It also acts as co-inducer of the transcriptional regulator PqsR (Xiao et al., 2006). We found that hydropriming significantly increased 2-Heptyl-4(1H)-quinolone in maize roots under salt stress.

2-Hydroxycinnamic acid is a derivative of hydroxycinnamic acids, which is involved in the regulation of plant growth and development and response to environmental stress (Luo et al., 2009). We found that that hydropriming significantly increased 2-Hydroxycinnamic acid in maize roots under salt stress.

Lupinine is a piperidine alkaloid whose concentration increases in plants under stress situations (Gill and Tuteja, 2010). We found that hydropriming significantly increased piperideine and lupinine in maize roots under salt stress.

2-Methylglutaric acid is the precursor of acetic acid (Scott, 1967). Previous research indicates that acetic acid priming may mitigate salt stress to plants by modulating lipid metabolism (Hu et al., 2021). We found that hydropriming significantly increased 2- Methylglutaric acid in maize roots under salt stress.

Plant growth regulators play critical roles in regulating plant responses to stress at extremely low concentration (Fariduddin et al., 2019). Melatonin is regarded as a candidate phytohormone that affects responses to biotic and abiotic stresses (Li et al., 2019). Rapid accumulation of melatonin in plants enhances salt resistance *via* its actions on antioxidants, photosynthesis, ion regulation and stress signaling (Li et al., 2019). Present study indicated that melatonin increased in salt-stressed and hydroprimed maize roots, which is agreement with previous result of Li et al. (2019).

*Caffeic acid 3-O-methyltransferase*(*COMT*) encodes caffeic acid O-methyltransferase, which is a multifunctional enzyme responsible for lignin and flavonoid biosynthesis (Kim et al., 2006). Caffeic acid O-methyltransferase can catalyse *N*-acetylserotonin into melatonin, suggestive of alternative melatonin pathways in plants (Chang et al., 2021). In present study, melatonin increased in salt-stressed and hydroprimed maize roots, while the expression of *COMT* remained unchanged in salt-stressed roots and increased in hydroprimed maize roots. Present study suggested the gene products (caffeic acid O-methyltransferase) is not the rate-limiting enzyme for melatonin synthesis, which is in accord with the result of Byeon et al. (2015).

Caffeic acid O-methyltransferase is also known as flavone 3’-O-methyltransferase and responsible for flavonoid biosynthesis. Flavone 3’-O-methyltransferase can transfer the methyl group specifically to the 3’-hydroxyl group of quercetin and luteolin, resulting in the formation of 3’-O-methylquercetin and 3’-O-methylluteolin (chrysoeriol). 3’-O-methylquercetin is further methylated to 3,7-Di-O-methylquercetin and ayarin (3,7,4’-Tri-O-methylquercetin) (Kim et al., 2006). In present study, 3’-O-methylquercetin remained unchanged in salt-stressed roots and decreased in hydroprimed maize roots. 3,7-Di-O-methylquercetin and ayarin increased in salt-stressed roots and decreased in hydroprimed maize roots. Chrysoeriol increased in salt-stressed and hydroprimed maize roots. While the expression of *COMT* remained unchanged in salt-stressed roots and increased in hydroprimed maize roots. Present study suggested flavone 3’-O-methyltransferase is not the rate-limiting enzyme for ayarin and chrysoeriol synthesis. The increase of chrysoeriol in hydroprimed maize roots may be related with increased expression of *Flavone 3’-O-methyltransferase* (*Caffeic acid O-methyltransferase*).

The alteration of endogenous levels of gibberellins is regulated by both developmental and environmental stimuli (Ryu and Cho, 2015). The triggering of gibberellins by salinity was depending on NaCl dosage, Gibberellins 8 was down accumulated at lower dosage and up accumulated at high dosage (Benjamin et al., 2019). Present results indicated that Gibberellins 8 increased under salt stress, which was in accord with previous results of Benjamin et al. (2019). Present study also indicated that Gibberellin A8 increased in hydroprimed roots, which is in accord with Ryu and Cho (2015) who found that GA application could be helpful to improve crop yields under salt stress condition.

Estrone is a steroid estrogen, which inhibit plant growth at high levels and promoted plant growth at low levels (Adeel et al., 2018). Present results indicated estrone decreased in salt-stressed and hydroprimed samples, which means maize adapted to salt stress by reducing estrogen content and hydropriming treatment promotes growth of maize under salt stress by reducing estrogen content. Present results is in agreement with the discover of Adeel et al. (2018).

Abscisic acid(ABA) is a phytohormone enabling plants to survive salt stresses (Costa et al., 2007). Cramer and Quarrie (2002) observed a negative relationship between leaf growth and ABA concentration under salt stress. Costa et al. (2007) observed that higher ABA is related to increased resistance to salt stress. The difference between the two results could attribute to differences in experimental methodology and differences in the range of ABA concentrations observed. Costa et al. (2007) spectulated that the growth-promotiing effect of low concentration ABA in plants under salt stress was dominant over its growth-inhibiting effect. Present results indicated ABA decreased in salt-stressed and hydroprimed samples, which means maize adapted to salt stress by reducing ABA content and hydropriming treatment promotes growth of maize under salt stress by reducing ABA content. Present results is in agreement with the spectulation of Costa et al. (2007).

Previous study indicated that brassinolide affects potato root growth in a dose-dependent manner (Hu et al., 2016). Low brassinolide concentrations promoted root elongation and lateral root development, whereas high brassinolide concentrations restrained root elongation (Hu et al., 2016). Present study showed that brassinolide decreased due to salt stress and hydropriming treatment. We speculated that maize may promote root elongation by reducing brassinosteroids concentration.

As reported, steroid reductase DET2 catalyzes the conversion of campestanol to castasterone, a major rate-limiting step in brassinolide biosynthesis (Li et al., 2023). In present study, the expression of *steroid reductase DET2* remind unchange in salt stressed roots and significantly increased in hydroprimed roots. Castasterone, the product of steroid reductase DET2 decreased in salt stressed roots and significantly increased in hydroprimed roots. Therefore, we speculated that the decreased of brassinolide in salt stress and hydropriming treatment was due to its degradation.

## 5 Conclusions

Soil salinization can significantly hamper germination and growth of maize crops. To combat salt stress, maize increases SOD activity and accumulates soluble sugar, soluble protein and proline. We found that maize also adjusts its metabolism. Our metabolite analysis indicates that salt stress significantly increased the content of 1278 metabolites and decreased the content of 1044 metabolites. Ethisterone(progesterone), a steroid hormone, was the most important metabolite produced in the roots grown from unprimed seeds in response to salt stress. Pathway enrichment analysis indicated the flavone and flavonol biosynthesis, which relates to scavenging reactive oxygen species (ROS), was the only significantly metabolic pathway affected by salt stress and activated by salt stress. Hydropriming significantly alleviated the adverse effects of salt stress and enhanced germination and seedling growth of maize under salt stress. Hydropriming significantly increased CAT activity, soluble sugar and proline content, decreased H_2_O_2_ under salt stress. Hydropriming significantly increased the content of 873 metabolites and significantly decreased the content of 1313 metabolites compared to the control. 5-Methyltetrahydrofolate, a methyl donor for methionine, was the most important metabolite produced in the roots of hydroprimed samples in response to salt stress. None of metabolic pathways were significantly affected by hydropriming treatment. Our results not only verified the important roles of some metabolites in resisting salt stress, but also further evidenced that flavone and flavonol biosynthesis and plant growth regulator relate to salt tolerance. The functions of other contributory metabolites related to salt stress amelioration in hydroprimed crops are not yet clear and warrants further analysis.

## Data availability statement

The original contributions presented in the study are included in the article/**Supplementary Material**. Further inquiries can be directed to the corresponding author.

## Author contributions

EZ: Conceptualization, Investigation, Data curation. XZ: Investigation, Methodology. WW: Investigation, Methodology. YS: Investigation. XT: Investigation. ZC: Investigation. XM: Investigation. YZ: Investigation. YW: Investigation. ZF: Investigation. NR: Writing-review and editing. DO’C: Writing-review and editing. XC: Formal analysis, Writing-review and editing. MY: Funding acquisition, Supervision, Visualization, Conceptualization, Writing- review and editing. All authors contributed to the article and approved the submitted version.
