# Supplementary material for: Metabolomics reveals the response of hydroprimed maize to mitigate the impact of soil salinization
Source: Front Plant Sci. 2023 Jun 7;14:1109460. doi: 10.3389/fpls.2023.1109460 (PMC10282767; doi:10.3389/fpls.2023.1109460)
Supplement: Supplementary file 1 [file DataSheet_1.docx]

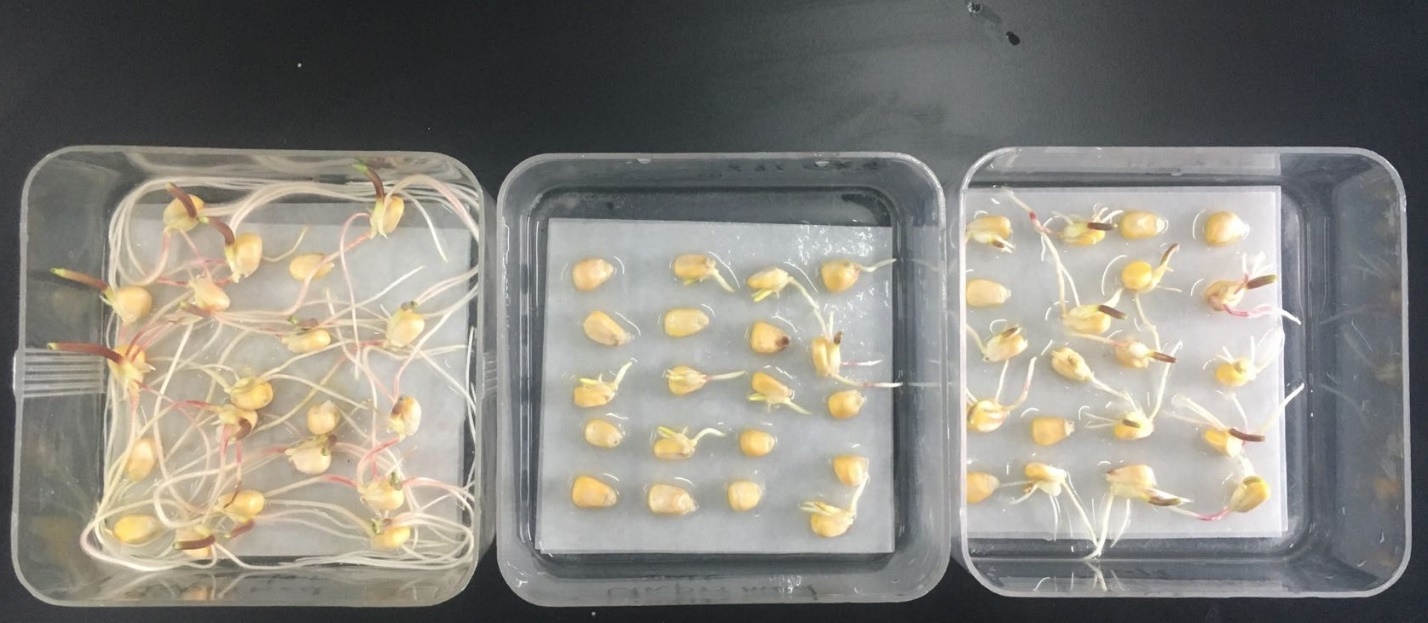


**Supplementary Figure 1**. Comparative germination of unprimed and hydroprimed samples under salt stress (NaCl) and control (unprimed water) on third day.


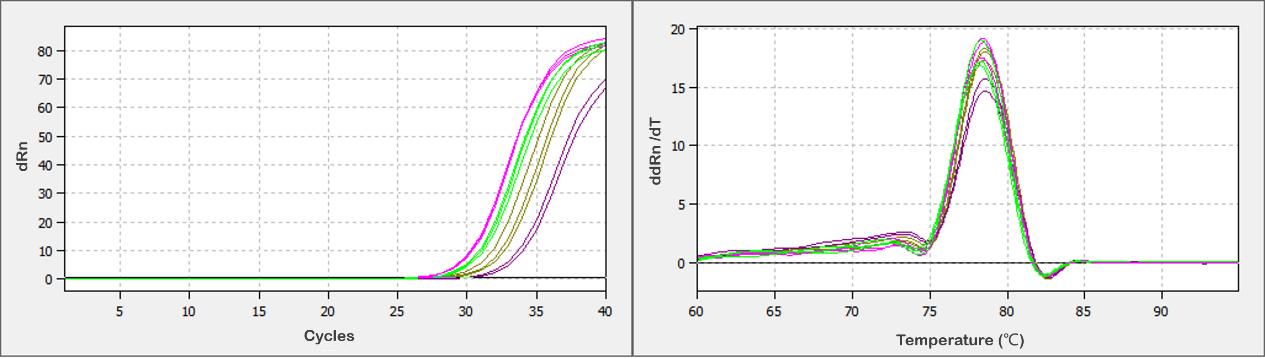


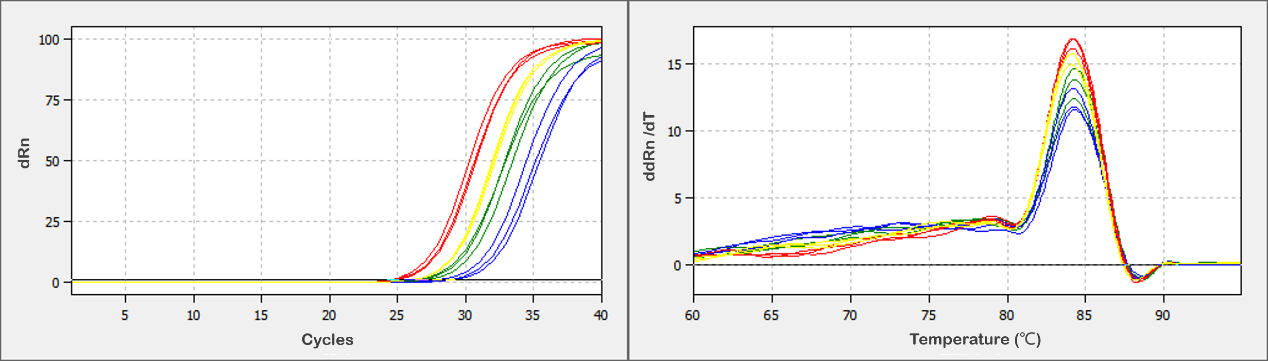


B1


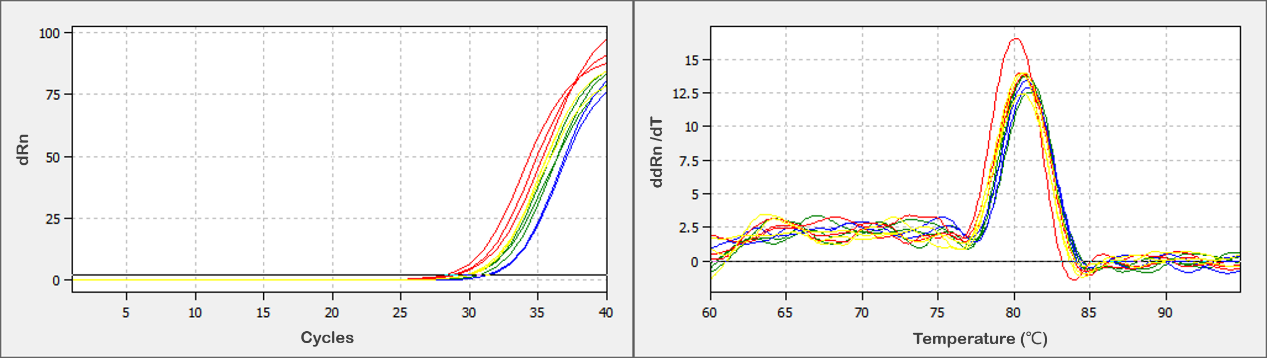


D2

D1

C1

C2


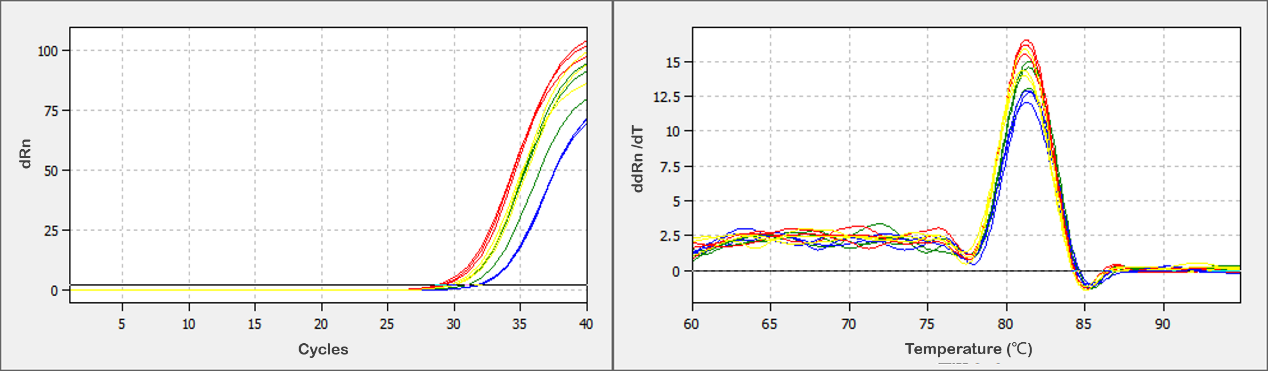


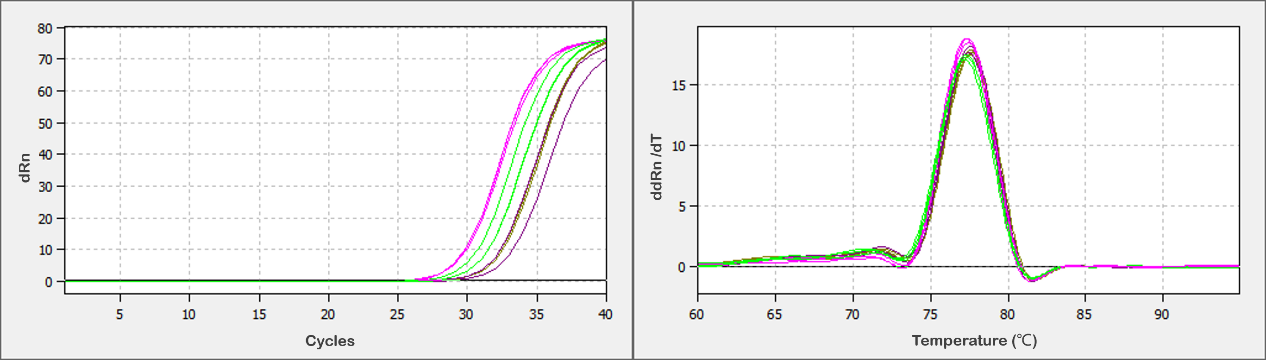


E2

E1

**Supplementary Figure 2** The amplification curve(A1, B1, C1, D1 and E1) and [solubility curve](C:/Program%20Files/Youdao/Dict/10.0.0.0/resultui/html/index.html#/javascript:;)(A2, B2, C2, D2 and E2) of *actin*, *delta-1-pyrroline-5-carboxylate synthase 2*, *caffeic acid 3-O-methyltransferase*, *catalase isozyme 2* and *steroid reductase DET2,* respectively*.*
