# Supplementary material for: Metabolomics reveals the response of hydroprimed maize to mitigate the impact of soil salinization
Source: Front Plant Sci. 2023 Jun 7;14:1109460. doi: 10.3389/fpls.2023.1109460 (PMC10282767; doi:10.3389/fpls.2023.1109460)
Supplement: Supplementary file 2 [file Table_1.doc]

Table 1 : Gene-specific primers used in qPCR.

| Gene | Primer Sequence (5′-3′)  (F, forward; R, Reverse) | Primers Tm (°C) |
| --- | --- | --- |
| *delta-1-pyrroline-5-carboxylate synthase 2（P5CS）* | F:TGGGGAGGCTCGGTTCTCTC | 61.45 |
|  | R:ACCAACAGCAGCACATGCCT | 57.35 |
| *caffeic acid 3-O-methyltransferase*  *(COMT)* | F:GTCATCGTCGTCGAGTGCGT | 59.40 |
|  | R:TAGGTGGCCTTGAACCCGGA | 59.40 |
| *catalase isozyme 2*  *(CAT2)* | F:CCGCTTCTCCACGGTGATCC | 61.45 |
|  | R:GCCGTCGCGGATGAAGAAGA | 59.40 |
| *steroid reductase DET2* | F:CGCTGTACCTCATCAGCCCG | 61.45 |
|  | R:GAGGGTGGGGCTCTCCATGA | 61.45 |
| *ZmActin2* | F:GCCATCCATGATCGGTATGG | 57.35 |
|  | R:GTCGCACTTCATGATGGAGTTG | 57.80 |
